# Learning under social versus nonsocial uncertainty: A meta‐analytic approach

**DOI:** 10.1002/hbm.25948

**Published:** 2022-05-27

**Authors:** Mario Martinez‐Saito, Elena Gorina

**Affiliations:** ^1^ Institute of Cognitive Neuroscience, HSE University Moscow Russia; ^2^ Department of Cognitive and Brain Sciences Hebrew University of Jerusalem Jerusalem Israel

**Keywords:** functional magnetic resonance imaging, iterated games, reinforcement learning, theory of mind

## Abstract

Much of the uncertainty that clouds our understanding of the world springs from the covert values and intentions held by other people. Thus, it is plausible that specialized mechanisms that compute learning signals under uncertainty of exclusively social origin operate in the brain. To test this hypothesis, we scoured academic databases for neuroimaging studies involving learning under uncertainty, and performed a meta‐analysis of brain activation maps that compared learning in the face of social versus nonsocial uncertainty. Although most of the brain activations associated with learning error signals were shared between social and nonsocial conditions, we found some evidence for functional segregation of error signals of exclusively social origin during learning in limited regions of ventrolateral prefrontal cortex and insula. This suggests that most behavioral adaptations to navigate social environments are reused from frontal and subcortical areas processing generic value representation and learning, but that a specialized circuitry might have evolved in prefrontal regions to deal with social context representation and strategic action.

## Graphical abstract

Much of the uncertainty that clouds our understanding of the world is caused by other people's actions and values. So there could be specialized mechanisms that compute learning signals under uncertainty of exclusively social origin operate in the brain. Our results suggest that most, but not all, behavioral adaptations to navigate social environments are reused from generic value representation and updating mechanisms.

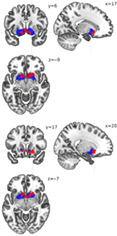

## 1 INTRODUCTION

Knowing what to do next is easier when we do not need to guess what other people are thinking. But in our increasingly interconnected society, there are fewer and fewer actions we can take which do not involve assessing how the presence of others will affect the outcome of our actions on the current state of affairs. Predicting the future requires guessing the present, but a substantial part of the relevant present is private to each of us (e.g., others' intentions or mood). Thus, most decisions, such as saying what you think to a colleague or keeping it to yourself, or investing your savings in biotech stocks or real state, are fraught with uncertainty that is compounded by social factors. Understanding to what extent learning in social contexts is a specialized function is important not only because we could expedite adaptation to different social contexts, but also because this would shed light on the source of cognitive biases, many of which are grounded on social preferences. In this study, we inquire into whether there are learning mechanisms specialized in resolving uncertainty of exclusively social origin.

Stochastic outcomes of know (risk) and unknown (uncertainty) probabilities can both be subjectively perceived as risk, and risk is a major modulating factor in learning (Niv et al., 2012; O'Neill & Schultz, 2010). Although uncertainty is a pervasive feature of the world, it is conspicuously higher in social environments of cooperating individuals, which characterize much of human evolution, than in nonsocial natural environments, which are more predictable because probabilities are comparatively easier to estimate. High uncertainty has even been posited to be a distinguishing feature between social and nonsocial environments (Mitchell, 2009). Thus, it would not be surprising that specialized mechanisms for learning under social uncertainty or noise were in place in the brain, which could have driven the encephalization of human ancestors (Schoenemann, 2006; Shultz & Dunbar, 2010). This is the social brain hypothesis (Ruff & Fehr, 2014), and it is congruent with reports of social interactions being functionally localized in the brain (Carter et al., 2012) and agents (Baez‐Mendoza et al., 2013), in the perception of faces and bodies (Allison et al., 2000; Peelen & Downing, 2007), and in social cognition (Amodio & Frith, 2006).

Adaptive behavior rests on appropriately assigning value to states and actions (Gold & Shadlen, 2007). This requires an elaborate internal representation of the world, which includes conspecifics. Crucially, mapping internal states and their values to actions is in general an intractable task which is greatly exacerbated by the need to predict the behavior of conspecifics (Frith & Frith, 2012; Yoshida et al., 2008). Since humans are conspicuously social animals, it is plausible that they evolved specific mechanisms to approach the problem of efficiently deriving reliable values from social interactions (Fletcher & Carruthers, 2012). This study attempts to elucidate to which extent the functional aspects of value learning in social contexts, specifically through social interactions, can be demarcated in the brain from value learning based on simple stimulus–reward contingencies. In other words, we ask whether learning and reward signals in social contexts generated by specialized, domain‐general, or overlapping circuits (FeldmanHall & Shenhav, 2019). This question can be operationalized by investigating, by way of a meta‐analysis of fMRI studies, whether the social aspect of value learning is functionally specialized enough to warrant functional segregation (Zeki & Shipp, 1988) within the neural substrates involved in general learning.

In particular, we investigate learning processes which involve updating state variables, such as values, through information acquired by way of repeated observation of other's actions, as opposed to through verbal or any kind of explicit instructions. These are typically behaviorally relevant variables—internal or derived from any sensory mode—that can be estimated as a statistic (in practice, the mean) of a univariate probability distribution. This includes reward/subjective utility, monetary value, appetitive and/or aversive states (Seymour et al., 2005), unsigned reward/saliency (Metereau & Dreher, 2013), probabilities of extrinsically non‐rewarded stimuli (Rodriguez et al., 2006), choice probabilities of other persons (Vanyukov et al., 2019), observational (as opposed to experiential) learning (Dunne et al., 2016), classical conditioning of items with emotionally salient others (Bray & O'Doherty, 2007; Watanabe et al., 2013), trustworthiness, and so on. These variables underpin value‐based decision making in a wide range of situations, typically in the context of a time‐evolving stochastic learning process, and in particular in learning process where the added uncertainty of social intermediaries hinders precise estimation of state values. Here, we will focus on the effects of uncertainty that has a social component on learning (e.g., emotional faces modulating learning of aversive signals; Robinson et al., 2013). This occurs, for example, in observational learning, and in any situation that requires deploying abilities pertaining to theory of mind (Yoshida et al., 2008), such as learning to predict other's preferences and behaviors. Knowing whom to trust (King‐Casas et al., 2005), financial markets (Lohrenz et al., 2007; Burke, Tobler, Baddeley, et al., 2010; Burke, Tobler, Schultz, et al., 2010), beauty contests (Coricelli & Nagel, 2009), poker, and in general any dynamically evolving game as long as it involves learning from other's actions or assessing the “social temperature” of groups of conspecifics, hinge on such learning processes.

Among theories devoted to explaining how people learn to behave efficiently and negotiate uncertainty, reinforcement learning (RL) is perhaps the most prominent and researched. The keystone of the theoretical framework built around RL is arguably temporal difference (TD) learning, which is an algorithm that allows agents act based on the future state values their actions lead to, by bootstrapping itself using current estimates. State value updates are accomplished by considering the difference between the expected reward and the actual received reward, where expectations account for all possible future states (Kishida & Montague, 2012): $V\left(s_{t+1}\right)=V\left(s_{t+1}\right)+\alpha \epsilon ,\epsilon =R_{t}-V\left(s_{t}\right)$, where *ε* is a (prediction) error signal, *ɑ* denotes learning rate, *R*_*t*_ is reward received by the agent as it moves into state *S* at time *t*, and *V*(*s*_*t*_) represents value function over states s_*t*_ at time *t*. Q‐learning extends the TD learning algorithm from values associated with states *V*(*s*_*t*_) to joint action‐value pairs (*s,a*) to which a value is assigned through a *Q = Q*(*s,a*) function that is updated upon feedback. Action selection is accomplished through a policy function that typically assigns higher choice probability to action‐values with high *Q*, such as the max or softmax functions. Q‐learning is relevant because it subsumes state and action value learning, thus furnishing a unifying simple and locally implementable algorithm that treats action‐state values as the learned unit. For our purposes, this enables looking upon actions and states on equal footing, and modeling the brain decision‐making mechanism as a putative implementation of TD learning. A further generalization of adaptive learning theories comes forth by considering off‐policy counter‐factual signals (fictive errors, e.g., Lohrenz et al., 2007), as opposed to experience‐based update of values. This further comprises situations where the subjective assessment of the missed reward for all the actions not taken is used postfact to update learned values, which it is likely to occur in human learning, as evinced by feelings of regret. This allows to use prediction error (PE) signals from outcomes that were not chosen, which in turns makes using RL in observation learning situations straightforward. A plethora of research has consistently shown that PE signals are associated with projections from dopaminergic neurons in the midbrain into the striatum (Schultz, 2016; Schultz et al., 1997). This crucial finding affords a means to test adaptive learning theories, such as RL, by combining fMRI data with behavioral modeling. Because of this direct and plausible link to brain activity, its simplicity and versatility, RL—and more generally adaptive learning—is posited to be one of three main components of social uncertainty resolution, along with automatic inference (such as impression formation) and purposeful deployment of executive control (FeldmanHall & Shenhav, 2019). This, along with the abundance of fMRI studies modeling learning with RL provides a rich testing ground for our research question.

Interactive play games (e.g., Hampton et al., 2008; Sanfey et al., 2003; Yoshida et al., 2008) offer a fruitful setting for comparing nonsocial with social learning of valuation. This setting provides a simple way to control the variables that govern the specification of values and uncertainties, thereby allowing to model learning with algorithms such as RL. Moreover, it also expedites the complex task of pinpointing which attributes of learning are most susceptible to biases or of social nature, through the application of theory of mind (Coricelli et al., 2009; Hampton et al., 2008; Yoshida et al., 2008). Since the advent of functional magnetic resonance imaging (fMRI), there has been a boom of studies devoted to the understanding of the neural mechanisms of decision‐making in social contexts, that combine fMRI with interactive play games. Thus, judicious selection of these studies should afford new insights into the degree to which social learning is specialized, and a better understanding of this process in turn would allow to better predict how learning is biased by social preferences and social uncertainty.

To some extent, social learning and general reward‐based learning rest on the same associative processes. General domain, higher‐level, value‐based decision‐making is subserved by a distributed network encompassing at least ACC, medial prefrontal cortex (mPFC), and striatum (Hampton & O'Doherty, 2007; Rushworth et al., 2007). However, similarly to expected values and rewards, PEs can also be functionally segregated by stimulus type, including by their sociality. For example, Valentin and O'Doherty (2009) reported evidence for at least partial functional segregation for some types of appetitive reward (money and juice) within the striatum. In general, midbrain neurons projecting through the mesolimbic pathway into the ventral striatum account for most of the activity associated to PEs, but there is also meta‐analysis evidence that specific reward types can differentially engage other regions such as operculum and insula for social rewards, amygdala for Pavlovian conditioning, and caudate for instrumental learning (Chase et al., 2015). Thus, there is some evidence pointing to a dissociation of social and nonsocial learning signals by reward type within the striatum, and by whether PEs are based on direct (unconditional) reward, or on variables with indirect value—such as observational learning—within the PFC. But at the same time, the striatum (Rilling et al., 2004) and other areas that enable value‐based decision making are recruited as well in social decision‐making (Rilling & Sanfey, 2011) and it is still unclear whether dissociations are induced by the source of uncertainty (social vs. nonsocial) as opposed to by value type or context.

In this study, we ask whether uncertainty of social origin induces functional segregation of signed PE signals, in the particular case where that such uncertainty is the result of learning through mediation of others, whether this occurs through direct mediation or observation. Since our main focus is learning of values, our main operational variable is the signed PE, whose main neural substrate is the striatum. In contrast, unsigned PEs are more related to saliency and social conflict than to learning, and are typically localized in anterior cingulate cortex (ACC) and insula (Baumgartner et al., 2009; Fukuda et al., 2019; Levorsen et al., 2021). More specifically, we focus on the role of the uncertainty attending social contexts in learning from error signals, but not on whether the learned values themselves are considered social values or not. We can assess how the incorporation of social uncertainty changes general reward‐based value learning, by analyzing the covariance of brain activity with feedback and error‐signaling events under different sources of uncertainty.

**FIGURE 1:**
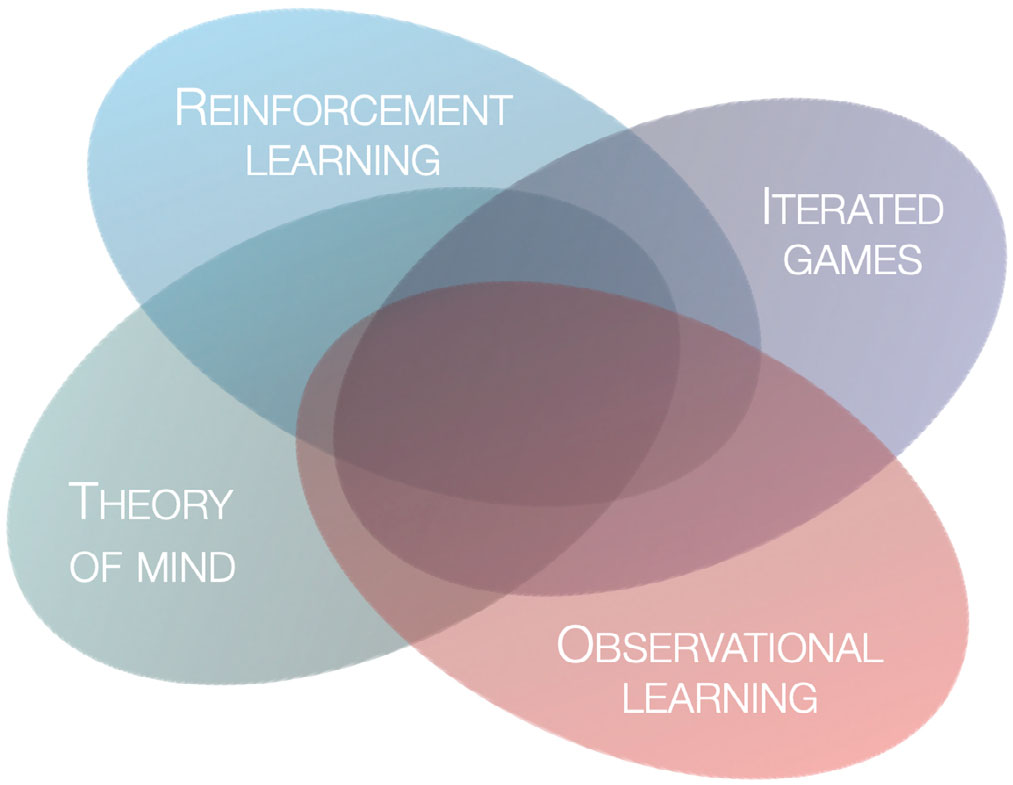
The meta‐analysis focuses on the functional specialization of learning mechanisms in the brain under uncertainty of social and nonsocial sources. This lies at the intersection of the fields of reinforcement learning, observational learning, theory of mind, and iterated game play

In summary, we focus on a type of learning that lies at the intersection of RL, observational learning, theory of mind, and iterated game play (Figure 1). We searched in academic databases and selected fMRI studies that comprised such learning tasks; and used them to perform a meta‐analysis on the location of activated foci in the brain. The goal of fMRI meta‐analyses is identifying areas of the brain that show consistent activation across studies and conditions, which allows to make inferences about differences in brain activity between learning conditions under social and nonsocial uncertainty. In contrast to similar previous fMRI meta‐analyses, which focused on general domain subjective value representation and expected values (Bartra et al., 2013; Clithero & Rangel, 2013; Gu et al., 2019), reinforcement learning signals across different reward types and contexts (Chase et al., 2015), social conformity signals (Wu et al., 2016), and the dissociation between PE valence and surprise (Fouragnan et al., 2018), here we aimed at learning signals, derived from any adaptive learning model, about a single signed variable, to study the effect of social uncertainty on learning.

## 2 METHODS

To assess differences between learning under social and nonsocial contexts, we classified the studies into two categories: “Social” and “Nonsocial,” where “Social” denotes a task where learning was accomplished in a social environment characterized by social proxies (other persons or surrogates) or interactions; “Nonsocial” included the remaining studies, that satisfied the eligibility criteria. We gathered PE and expected or predicted value maps for both categories (SPE for social PE; NPE for nonsocial PE), where PE was defined as any learning signal; however, only PE maps were used in the analyses because expected value maps were not consistently reported and its number was insufficient.

### 2.1 Data collection and study selection criteria

**FIGURE 2:**
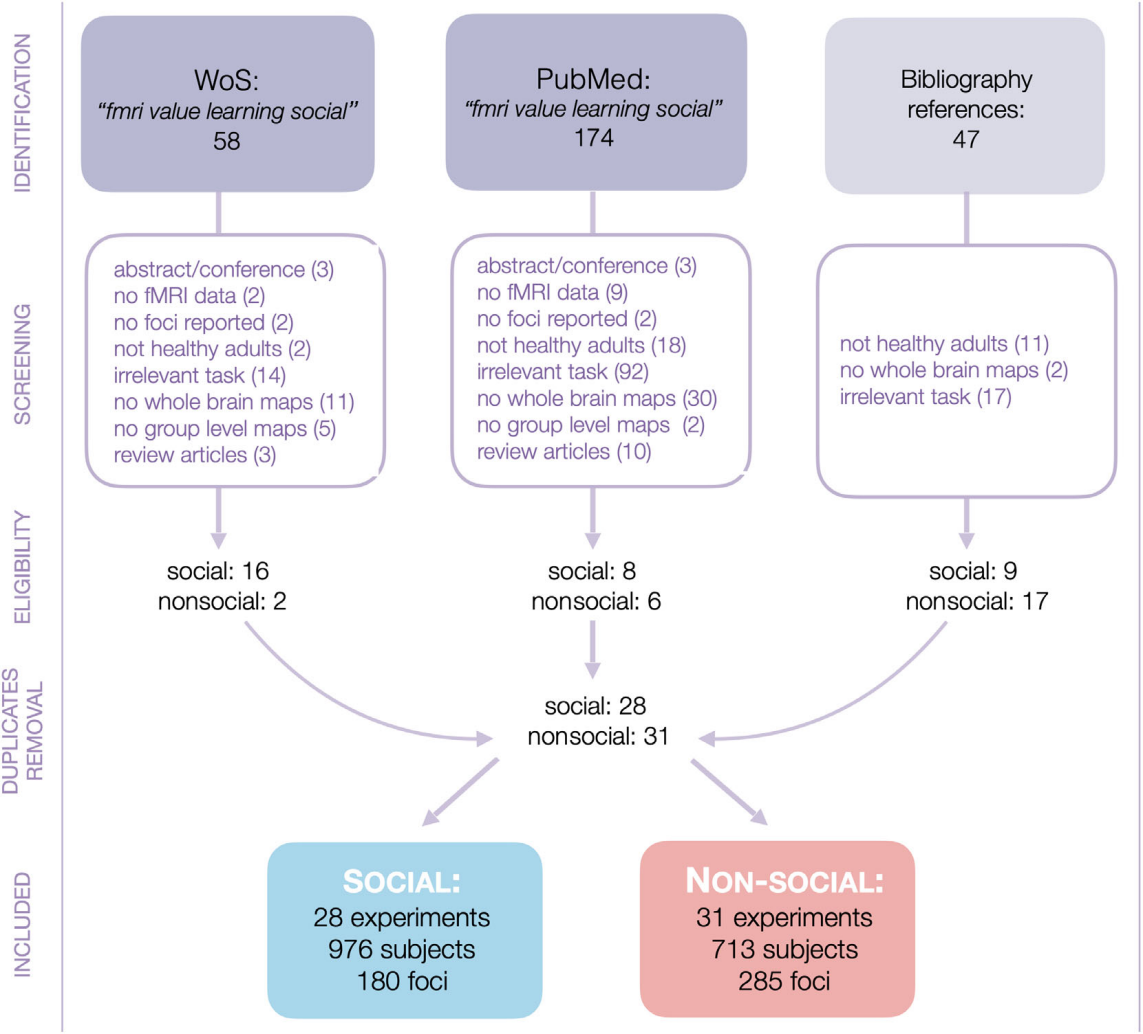
Scheme of the process used to identify and cull articles that met the criteria of the two groups used in meta‐analyses, in PRISMA flowchart format (Moher et al., 2009). Some studies count as both social and nonsocial

We searched for studies through the websites PubMed and Web of Science, which aggregate multiple academic databases, on March 3, 2021. The keywords used to filter results were: (“fmri” AND “learning” AND “social”). We selected all accessible articles that satisfied the inclusion criteria. In brief, the selection consisted of two stages: identification, or abstract‐based culling, and screening, where studies lain aside during identification are perused to checked all inclusion criteria (Figure 2). We also found studies by tracing citations of discovered articles and references in the bibliography of reviews. If whole‐brain maps were not included in an article that satisfied the remaining requirements, we selected it in case we could retrieve the maps by contacting the authors. Although the keywords included the term “social,” we found enough studies to conduct meta‐analyses for both categories. When the statistical significance level reported in a study was unclear, we followed the recommendations of Albajes‐Eizagirre et al. (2019, section 4.3). The selected articles satisfied the following criteria:

- Participants were healthy adult humans within the age range 18–65 years, in nonclinical studies.
- Whole‐brain results were reported. Studies that only reported region of interest (ROI) or small volume correction (SVC) analyses were excluded, unless their peak activation statistics exceeded the threshold used for whole‐brain analysis. This is akin to simulating a more conservative (whole‐brain) threshold being applied to subregions (Müller et al., 2018).
- The task was a repeated and continuous learning paradigm where every trial comprised a stimulus presentation upon which a prediction was made, a choice, and feedback. Learning should not involve more than one variable during the whole task. Thus, participants are required to monitor, learn, and act on one single variable.
- The study included an adaptive learning model which enacts behavior, typically a RL algorithm, but not restricted to it. RL algorithm derivatives adapted to monitoring not only values, but also choice or behavior probabilities and other experiential or observational values such as trustworthiness, salience, or risk were considered as long as the learning algorithm employed a PE‐like computed as some monotonic function of the difference between a prediction and the actual outcome. This is because we focus on learning‐related activations, a not on RL per se. The variables being predicted could represent behaviorally relevant quantity, such as values, of stimuli, states, actions, or even statistics (in practice, mean or variance/risk) of probability distributions, whether these referred to self or to other's attributes. This is deliberately encompassing to allow the inclusion of a large enough cohort of studies. The study should also have used these PE‐like variables as parametric modulators for the relevant task events—typically feedback—in the design matrix of a general linear model analysis of BOLD signal. Maps of variables anti‐correlated with PEs were excluded, that is, we used maps correlated with signed PE.
- Additionally, only for the “Social” category (26 studies found), the learning task should occur only through repeated observation of other person's actions, and for the “Nonsocial” category (30 studies), it should exclude such social proxies.

In total, we selected 50 studies, that included 28 experiments with SPE maps (180 foci, 976 subjects) and 31 experiments with NPE maps (285 foci, 713 subjects). A comprehensive list is shown on Table 1.

**TABLE 1:** The selected articles, with the type of learning signal maps included in each. When not explicit, cluster‐level corrections are based on SPM's (Friston et al., 1995) Gaussian random field theory implementation (Friston et al., 1994). When an article is found in multiple sources, bibliography references are listed preferentially instead of search engines. The source can be either a database search engine or a bibliography.

	Authors	Number of subjects	Number of foci	Type of map	Most liberal threshold	Source	Notes (description of PEs, PEs maps used, etc.)
1	Apps et al. (2015)	16 (10 female)	1	SPE	Voxel‐wise *z* >3.17, cluster FDR *p* <.05	WoS	
2	Behrens et al. (2008)	24 (10)	6	SPE	Voxel‐wise *z* >3.5, nvox >50	Campbell‐Meiklejohn et al. (2017)	
			10	NPE			
3	Boorman et al. (2011)	19 (10)	8	NPE	Voxel‐wise *p* <.001, nvox >10	WoS	Here, both experiential and counterfactual choices generate PE.
4	Boorman et al. (2013)	25 (8)	2	SPE	Voxel‐wise *z* >3.1, nvox >10	Collette et al. (2017)	
			7	NPE			
5	Bray and O'Doherty (2007)	22 (13)	6	SPE	Voxel‐wise *p* <.0001, nvox >5	Chase et al. (2015)	SPE: attractive‐unattractive conditioning; pairing of items with other persons.
6	Brovelli et al. (2008)	14 (7)	2	NPE	Voxel‐wise?, *p* <.01 FWER, nvox >5	Chase et al. (2015)	NPE: visuomotor learning. We used only the signed (positively correlated) PE.
7	Campbell‐Meiklejohn et al. (2010)	28 (13)	5	SPE	*z* >2.3, cluster FWER *p* <.05 (FSL default)	PubMed	SPE: review outcome (agreem > disag). NPE: object outcome (pref > nonpref).
			5	NPE			
8	Chien et al. (2016)	32 (16)	2	NPE	Voxel‐wise *p* <.001	PubMed	SVC foci excluded: none. (using as threshold the t‐statistic [3.78] of the lowest significant peak at whole‐brain *p* <.001).
9	Christopoulos and King‐Casas (2015)	72	5	SPE	*p* <1e−6, nvox >5	WoS	SPE: other‐related PE. NPE: self‐related PE.
			9	NPE	*p* <1e−4, nvox >5		
10	Collette et al. (2017)	50 (25)	7	SPE	Voxel‐wise *p* <.001, cluster FWER *p* <.05	PubMed	SPE: agent‐referential PE.
11	Cooper et al. (2014)	38 (18)	3	SPE	Voxel‐wise *p* <.001, cluster FWER *p* <.05	WoS	SPE: violation of expectations in dating.
12	Dunne et al. (2016)	23 (10)	2	SPE	Voxel‐wise *p* <.005, cluster FWER *p* <.05	WoS	SPE: state prediction errors (StatePE) used in observational learning. NPE: reward PE experienced.
			5	NPE			
13	Evans et al. (2011)	18 (6)	4	SPE	Voxel‐wise *p* <.005, nvox >30, cluster FWER *p* <.05	WoS	
14	Fareri et al. (2012)	18 (9)	6	SPE	Cluster FWER *p* <.05 from the (Monte Carlo) cluster level statistical threshold estimator plugin in BrainVoyager (voxel‐wise *p* <.001, nvox >7)	Chase et al. (2015)	
15	Garvert et al. (2015)	29 (14)	1	SPE	Voxel‐wise *p* <.01, cluster FWER *p* <.05	WoS	SPE: PE as surprise.
16	Gershman et al. (2009)	16	2	NPE	Cluster FWER *p* <.05	Chase et al. (2015)	
17	Glaescher et al. (2009)	20 (9)	10	NPE	Voxel‐wise *p* <.001	PubMed	
18	Hampton et al. (2006)	16 (8)	3	NPE	Voxel‐wise *p* <.001	WoS	NPE: posterior minus prior correct probabilities.
19	Harris and Fiske (2010)	15 (4)	4	SPE	Voxel‐wise *p* <.001, nvox >10	WoS	SPE: pooled warmth and competence expectancy violation.
20	Howard‐Jones et al. (2010)	16 (6)	21	SPE	Voxel‐wise *p* <.001	Chase et al. (2015)	SPE: egocentric PE.
			20	NPE			
21	Jones et al. (2011)	46 (22)	4	SPE	Cluster FWER *p* <.05 from AFNI permutation tests (voxel‐wise *p* <.005, nvox >50)	PubMed	
22	Kahnt et al. (2009)	19 (10)	17	NPE	Voxel‐wise *p* <.001, nvox >15	Chase et al. (2015)	
23	Kahnt et al. (2012)	23 (10)	2	NPE	Cluster FWER *p* <.05	PubMed	
24	Klucharev et al. (2009)	21 (21)	12	SPE	Voxel‐wise *p* <.001, cluster FDR *p* <.05	Apps et al. (2015)	SPE: based on the degree of social conflict. SVC foci excluded: [10, –21, –14], [−3, –15, –3], and [3, –27, –3].
25	Levorsen et al. (2021)	25 (25)		SPE	Voxel‐wise *p* <.005, nvox >20	Other	SPE: based on the degree of social conflict.
				NPE			
26	Li et al. (2006)	46 (24)	7	NPE	Voxel‐wise *p* <.001, nvox >3	Chase et al. (2015)	
27	Lin et al. (2012)	27 (27)	3	NPE	Voxel‐wise *p* <.001, nvox >15	PubMed	NPE: in the “social” condition, sociality is irrelevant to learning, so it is considered as NPE.
28	Madlon‐Kay et al. (2013)	23 (17)	6	NPE	Voxel‐wise *p* <.005, cluster FWER *p* <.05	Chase et al. (2015)	SVC foci excluded: [−10, 8,–6], [10, 10,–8].
29	Martinez‐Saito et al. (2019)	27 (18)	10	SPE	Voxel‐wise *p* <.001	WoS	
30	Metereau and Dreher (2013)	20 (10)	18	NPE	Cluster *p* <.05 FDR	Chase et al. (2015)	NPE: it reflects a measure of PE saliency. We used only the largest group of foci (Gustatory) among all the reported (which were partially derived from the same data), to avoid correlations which could inflate significance.
31	Niv et al. (2012)	16 (4)	5	NPE	Cluster *p* <.05 FWER	Chase et al. (2015)	
32	Nook and Zaki (2015)	21 (18)	4	SPE	Cluster FDR <.05 from neuroelf's AlphaSim permutation test (*p* <.005, nvox >25)	WoS	SPE: disagreement > consensus.
33	O'Doherty et al. (2003)	13 (9)	20	NPE	Voxel‐wise *p* <.001	Chase et al. (2015)	NPE: map of jointly significant unconditioned (US) and conditioned stimuli (CS) regressors.
34	Payzan‐LeNestour et al. (2013)	17 (8)	44	NPE	Cluster FWER *p* <.05, from AFNI's AlphaSim (nvox >186)	PubMed	NPE: both expected and unexpected PEs, derived from a Bayesian model.
35	Robinson et al. (2013)	24 (16)	6	SPE	Voxel‐wise *p* <.005	Chase et al. (2015)	SPE: socially‐mediated stress effect on aversive PE (stress * valence interaction).
36	Rodriguez et al. (2006)	15	4	NPE	Voxel‐wise *p* <.01, cluster FWER *p* <.05	Chase et al. (2015)	NPE: includes both negative and positive feedback events.
37	Rodriguez (2009)	14 (8)	5	NPE	Voxel‐wise *p* <.005, nvox >5	Chase et al. (2015)	NPE: includes both unlearnable and learnable stimuli.
38	Schönberg et al. (2007)	29 (15)	15	NPE	Voxel‐wise *p* <.001, nvox >5	Chase et al. (2015)	
39	Seger et al. (2010)	10 (5)	16	NPE	Cluster FDR *p* <.05 with Brain Voyager	Chase et al. (2015)	
40	Seymour et al. (2005)	19	13	NPE	Voxel‐wise *p* <.001, nvox >5	Chase et al. (2015)	NPE: includes learning about both appetitive and aversive rewards.
41	Sul et al. (2015)	26 (26)	3	SPE	Voxel‐wise *p* <.001, nvox >15	PubMed	Although this study compares values about self and others, with no real feedback from others (only learning of probabilities), it is deemed as SPE because these probabilities bear on the valuation of others.
			1	NPE			
42	Suzuki et al. (2012)	36	8	SPE	Cluster FWER *p* <.05 from AFNI's AlphaSim (*p* <.005, nvox >56)	WoS	SPE: pooled simulated‐other's reward and action PEs.
43	Suzuki et al. (2016)	24 (10)	7	SPE	Cluster FWER *p* <.05 from AFNI's AlphaSim (*p* <.005, nvox >63)	WoS	SPE: interpreted as Kullback–Leibler divergence of the learning signal.
44	Takemura et al. (2011)	23 (8)	8	NPE	Voxel‐wise *p* <.001, nvox >5	Chase et al. (2015)	NPE: only the WITH model.
45	Tanaka et al. (2006)	18 (5)	7	NPE	Voxel‐wise *p* <.001, nvox >40	Chase et al. (2015)	NPE: pooled RANDOM and REGULAR treatments.
46	van den Bos et al. (2013)	25 (13)	2	SPE	Vowel‐wise?, nvox >10, cluster FWER *p* <.05	PubMed	
47	Vanyukov et al. (2019)	40 (25)	10	SPE	Voxel‐wise *p* <.0001, cluster correction permutation method 3dttest++ *p* <.05	WoS	SPE: based on expectations about others' policy.
48	Watanabe et al. (2013)	20 (10)	5	SPE	Voxel‐wise *p* <.001, nvox >15	Chase et al. (2015)	SPE: pairing of items with emotionally salient others.
49	Zaki et al. (2011)	14 (0)	11	SPE	Cluster FWER *p* <.05 from Monte Carlo simulation (*p* <.0005, nvox >25)	WoS	SPE: reaction to the faces of peers with varying social rating.
50	Zhang & Gläscher (2020)	39 (20)	3	SPE	Cluster FWER *p* <.05 for both cluster formation and correction	WoS	SPE: combination of directed learning and observational learning error signals.

Abbreviations: ?, datum not reported; FDR, false discovery rate correction; FWER, family‐wise error rate correction; nvox, number of voxels in cluster; SVC, small volume correction; WoS, web of science.

### 2.2 Meta‐analysis estimation

#### 2.2.1 Activation likelihood estimation

We accomplished a meta‐analysis of fMRI studies with GingerALE 3.0.2 (Eickhoff et al., 2012), which implements the activation likelihood estimation (ALE) method. ALE is a type of coordinate‐based meta‐analysis (CBMA, which uses solely coordinates of cluster peaks in statistical parametric maps) that is the most widely used approach for fMRI data meta‐analysis (Samartsidis et al., 2017). Although full statistic image data contains information that could dramatically improve meta‐analysis accuracy over peak foci data (Salimi‐Khorshidi et al., 2009), sharing of full data is rare; conversely, coordinate data is available in databases such as NeuroSynth (Yarkoni et al., 2011), which explains the prevalence of CBMA, whose flagship is ALE. ALE discards information contained in the *t*‐statistics and uses a mixture of Gaussian distributions discretized over voxels and centered around the reported foci locations to model the probability that specific voxels correspond to true activations. The variance of the Gaussian kernels was heuristically estimated empirically by Eickhoff et al. (2009) on a single study using 21 subjects. The images containing smoothed foci are called focus maps. The focus maps are combined into a single map of ALE statistics, which for every voxel represent the probability that at least one of the closest reported activations is truly located at the voxel (Turkeltaub et al., 2012). Statistical significance is assessed with a Monte Carlo permutation test under the null hypothesis of uniformly random spatial distribution between studies (Eickhoff et al., 2009). In particular, during sampling, the spatial arrangement of activations within a study is preserved to test for above‐chance clustering between studies. This enables modeling study identities as random effects and thus generalize results to the population. Eickhoff et al. (2016) recommend using at least 20 experiments per condition to achieve sufficient power for moderate effects with ALE.

In single dataset analyses, ALE images (the maps of ALE statistics) were thresholded with a cluster‐forming level of *p* <.001 uncorrected; the resulting ALE clusters were culled via cluster‐level inference with a family‐wise error rate (FWER) correction of *p* <.05, which was achieved by comparing the observed cluster size to a Monte Carlo maximum cluster size distribution built from 5000 iterations of drawing random peak locations from the gray matter template. Cluster‐level FWER correction is the most appropriate method for statistical inference to ALE (Eickhoff et al., 2016). Conjunction analysis use the voxel‐wise minimum statistic between two cluster‐thresholded ALE images. Subtraction (contrast) images are the result of subtracting one input image from the other and were thresholded at *p* <.001. Between‐group study size difference in subtraction analysis is accounted for using a permutation test (we ran 10,000) by pooling the foci datasets and assigning them randomly to a group (Eickhoff et al., 2012). Anatomical labeling was supported by the Talairach Daemon (Lancaster et al., 2000).

#### 2.2.2 Seed d‐Mapping with Permutation of Subject Images

CBMA methods—including ALE—test for spatial convergence of activation peaks. This roughly means that they detect regions where studies report more peaks or peaks are found more often than in other regions. But this contrasts with typical fMRI analyses, where massive univariate voxelwise tests detect voxels that activate, perhaps as differences between groups. ALE convergence of peaks test relies on spatial assumptions that data are not guaranteed to meet (voxels are independent and have the same probability to have a false peak, but in reality gray matter voxels covariate with their neighbors in way that depends on tissue composition), and has lower statistical power when there are multiple effects, which may undermine the statistical significance of the results (Albajes‐Eizagirre & Radua, 2018).

An alternative approach is to use the peak *t*‐statistics to estimate effect sizes, thus enabling random and fixed effects modeling, which increases reliability and accuracy (Bossier et al., 2018). By assessing the influence of the type of group‐level model used in studies (fixed effects, ordinary least squares, mixed effects), the type of CBMA (ALE, fixed effects, and random effects) and the amount of studies included in the analysis, Bossier et al. (2018) concluded that combining mixed effects models in the second stage of the GLM procedure with random effects meta‐analyses was optimal in terms of the type I versus type II error balance and activation reliability. Among the CBMAs that discard effect sizes, ALE is a good alternative in terms of the balance between type I and II errors, but it requires more studies (35, as opposed to 20 for random‐effects CBMAs) to achieve similar activation reliability (Bossier et al., 2018). The shortfalls of CBMAs, which discard most of the full statistical image information, can be partly alleviated by accounting for both activations and deactivations so that contradictory findings cancel each other (Radua & Mataix‐Cols, 2009), and through the use of effect sizes, that is, *z*‐ or *t*‐statistics (Radua et al., 2012). These two features, together with subject‐based permutation test to control FWER, feature in the algorithm Seed‐based d‐Mapping with Permutation of Subject Images (SDM‐PSI; Albajes‐Eizagirre, Solanes, Vieta, & Radua, 2019), which can thereby implement standard voxel‐wise tests. SDM‐PSI uses the AAL atlas for anatomical labeling (Tzourio‐Mazoyer et al., 2002).

We employed SDM‐PSI to probe the robustness of ALE results and to capitalize on its built‐in algorithms to grade the strength of evidence via multiple robustness considerations, viz. study heterogeneity (*I* ^2^ statistic), small‐study effect (metabias test, which assesses funnel plot asymmetry), and excess significance (which would indicate that more studies than expected report significant results). We performed voxel‐wise effect size meta‐analyses separately for the social and nonsocial conditions (similarly to ALE single dataset analyses). We also tested the (linear) effect of the dummy variable social versus nonsocial through a SDM‐PSI meta‐regression, which assessed the covariation of the predictor dummy variable {SPE = 1, NPE = 0} with voxel activations. The data were preprocessed with SDM‐PSI's (version 6.22) default gray matter correlation template (voxel size of 2 × 2 × 2 mm, isotropic Gaussian smoothing kernel FWHM of 20 mm). SDM‐PSI accomplishes FWER correction by running a subject‐base permutation test that yields a distribution of the maximum statistic that is used to threshold the meta‐analysis images. All models were estimated with 50 random imputations and statistical thresholding was performed through 1000 permutations using a voxel‐level FWE‐corrected threshold of *p* <.05.

## 3 RESULTS

**FIGURE 3:**
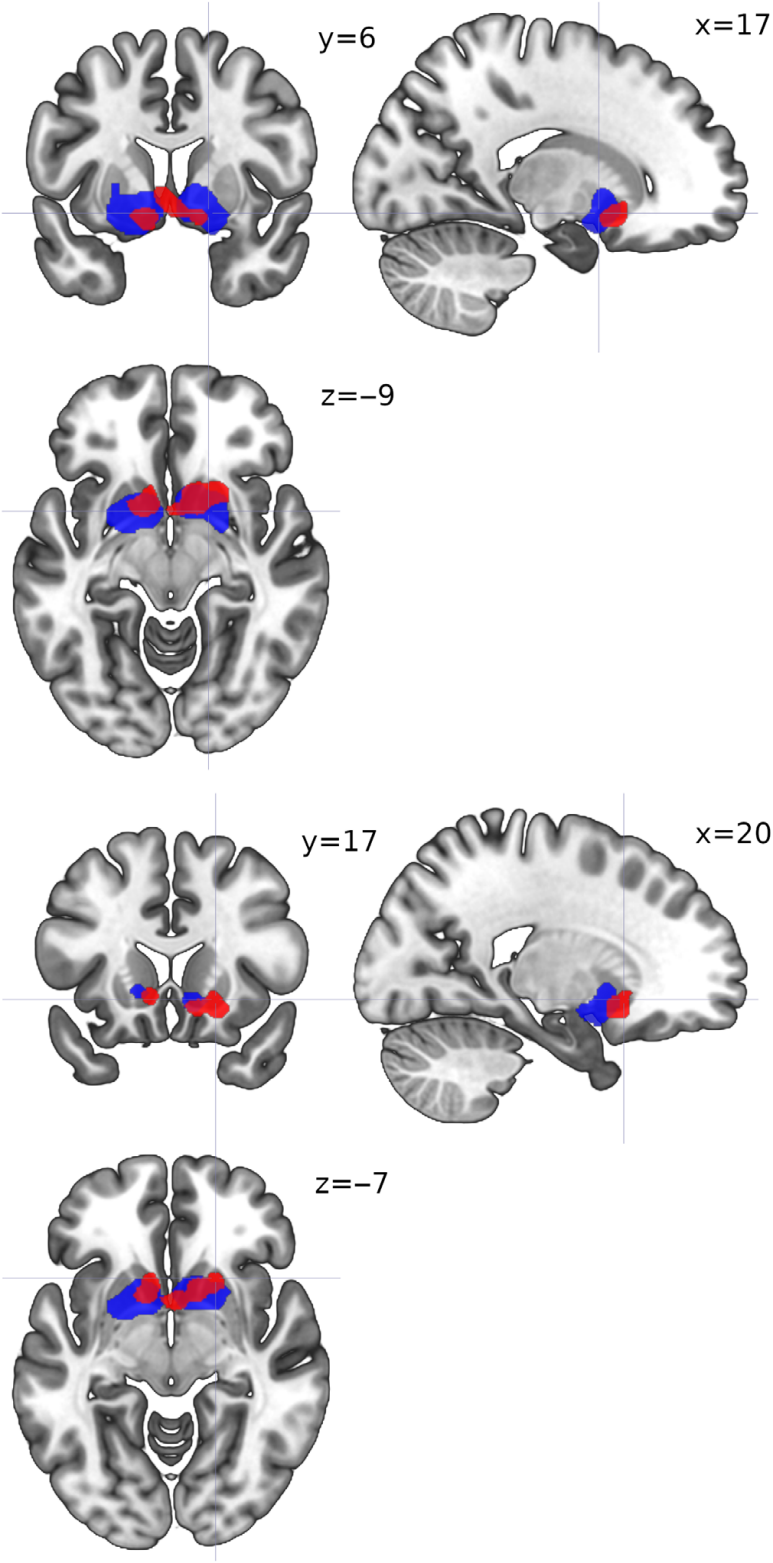
ALE image for social (red) and nonsocial (blue) error signal studies. Activation likelihood estimation maps were thresholded at *p* <.05. Images rendered by MRIcroGL (Rorden & Brett, 2000)

**FIGURE 4:**
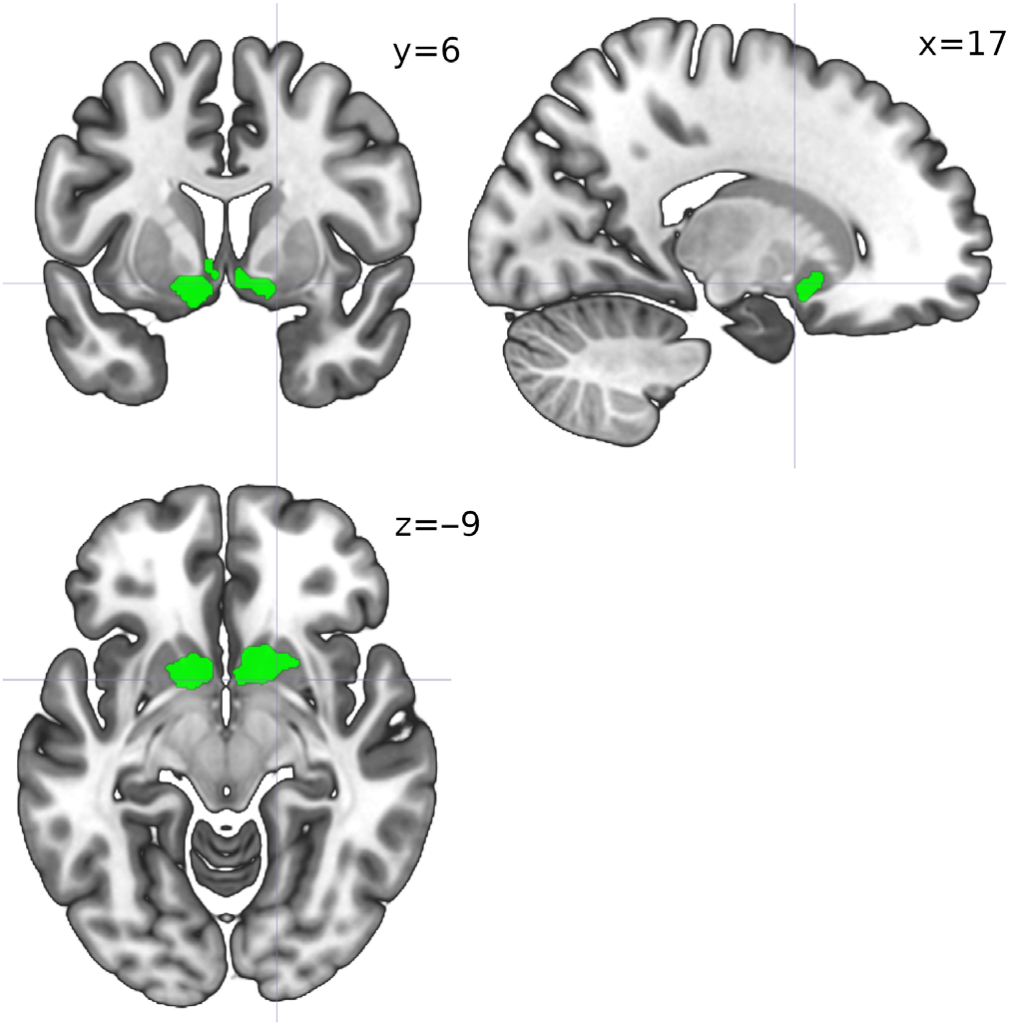
Conjunction activation likelihood estimation image between social and nonsocial groups

The ALE meta‐analysis found clusters in ACC and striatum for both social (SPE) and nonsocial prediction error signal (NPE) studies (Table 2, Figure 3). For SPE, two clusters with 4 and 2 peaks were found with the maximum located bilaterally in the putamen, and for NPE two clusters with 1 and 2 peaks each with the maxima in the right caudate head and the left lateral globus pallidus. SPE caudate head activations were bilateral but more rostral in the right hemisphere and more caudal in the left, whereas NPE striatal activations were distributed symmetrically and extensive, including caudate head, and the lentiform nucleus, which comprises the putamen and the globus pallidus (Figure 3, Table 2). Thus, striatal regions were more likely to be activated in NPE than in SPE experiments, but no regions were more likely to be activated in SPE than in NPE experiments (Table 2). The conjunction analysis yielded 2 clusters with 4 and 2 peaks (Figure 4, Table 2). These clusters indicate the location of the strongest correlate for both social and nonsocial learning signals.

**TABLE 2:** Surviving activation likelihood estimation (ALE) meta‐analysis clusters

Analysis type	Cluster anatomic location (% cluster volume)	Cluster size (mm^3^)	Peak coordinates (MNI)	Peak *z* statistic	Contributing studies
SPE group	Right cerebrum (63.3) Putamen (45.5) Caudate head (37.4) Lateral globus pallidus (5.9) Anterior cingulate, BA 25 (2.1)	2664	14, 10, −12	4.83	(Christopoulos & King‐Casas, 2015; Evans et al., 2011; Fareri et al., 2012; Howard‐Jones et al., 2010; Jones et al., 2011; Levorsen et al., 2021; Martinez‐Saito et al., 2019; van den Bos et al., 2013; Watanabe et al., 2013; Zhang & Gläscher, 2020)
	Left cerebrum (100) Caudate head (42.1) Putamen (23.0) Lateral globul pallidus (15.9) Medial globus pallidus (1.6)	1288	−10, 8, 12	4.81	(Bray & O'Doherty, 2007; Campbell‐Meiklejohn et al., 2010; Levorsen et al., 2021; Zaki et al., 2011; Zhang & Gläscher, 2020)
NPE group	Right cerebrum (100) Putamen (36.3) Caudate head (34.7) Lateral globus pallidus (3.7) Medial globus pallidus (2.9) Anterior cingulate, BA 25 (2.9) Caudate body (2.3)	4496	10, 10, −6	7.55	(Behrens et al., 2008; Boorman et al., 2011; Boorman et al., 2013; Campbell‐Meiklejohn et al., 2010; Christopoulos & King‐Casas, 2015; Dunne et al., 2016; Gläscher et al., 2009; Hampton et al., 2006; Kahnt et al., 2009; Lin et al., 2012; Li et al., 2006; Metereau & Dreher, 2013; Seger et al., 2010; Seymour et al., 2005)
	Left cerebrum (100) Putamen (53) Lateral globus pallidus (18.2) Caudate head (15.8) Medial globus pallidus (6.3) Entorhinal area, BA 34 (2.2)	5032	−12, 8, −8	6.95	(Boorman et al., 2013; Campbell‐Meiklejohn et al., 2010; Chien et al., 2016; Christopoulos & King‐Casas, 2015; Gläscher et al., 2009; Hampton et al., 2006; Howard‐Jones et al., 2010; Kahnt et al., 2009; Kahnt et al., 2012; Lin et al., 2012; Li et al., 2006; Metereau & Dreher, 2013; Niv et al., 2012; O'Doherty et al., 2003; Seger et al., 2010; Seymour et al., 2005; Sul et al., 2015; Tanaka et al., 2006)
Conjunction SPE * NPE	Right cerebrum (100) Caudate head (41.4) Putamen (37.8) Lateral globus pallidus (9.9) Anterior cingulate, BA 25 (3.6)	1376	12, 10, −12	—	(Boorman et al., 2013; Campbell‐Meiklejohn et al., 2010; Evans et al., 2011; Fareri et al., 2012; Gläscher et al., 2009; Levorsen et al., 2021; Martinez‐Saito et al., 2019; Seger et al., 2010; Seymour et al., 2005; Zhang & Gläscher, 2020)
	Left cerebrum (100) Caudate head (31.9) Putamen (29.7) Lateral globus pallidus (22.0) Medial globus pallidus (2.2)	984	−10, 8, −10	—	(Boorman et al., 2013; Campbell‐Meiklejohn et al., 2010; Christopoulos & King‐Casas, 2015; Kahnt et al., 2012; Levorsen et al., 2021; Lin et al., 2012; Zhang & Gläscher, 2020)
Subtraction SPE‐NPE	No clusters survived				
Subtraction NPE‐SPE	No clusters survived				

**FIGURE 5:**
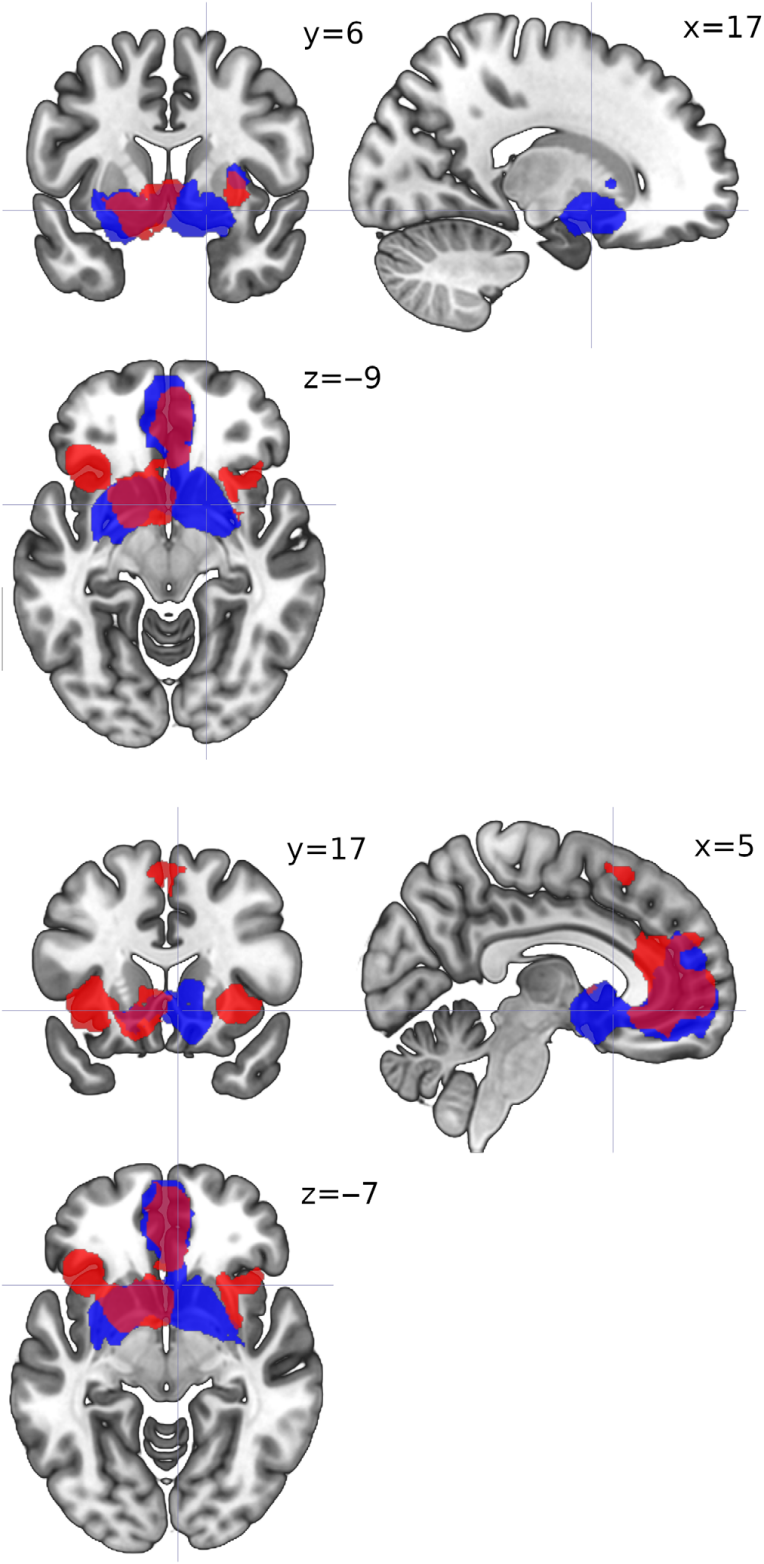
Activation maps for social (red) and nonsocial (blue) error signal studies (analogous to Figure 3) computed with Seed‐based d‐Mapping with Permutation of Subject Images (SDM‐PSI). Cluster‐level inference with family‐wise error rate of *p* <.05

**FIGURE 6:**
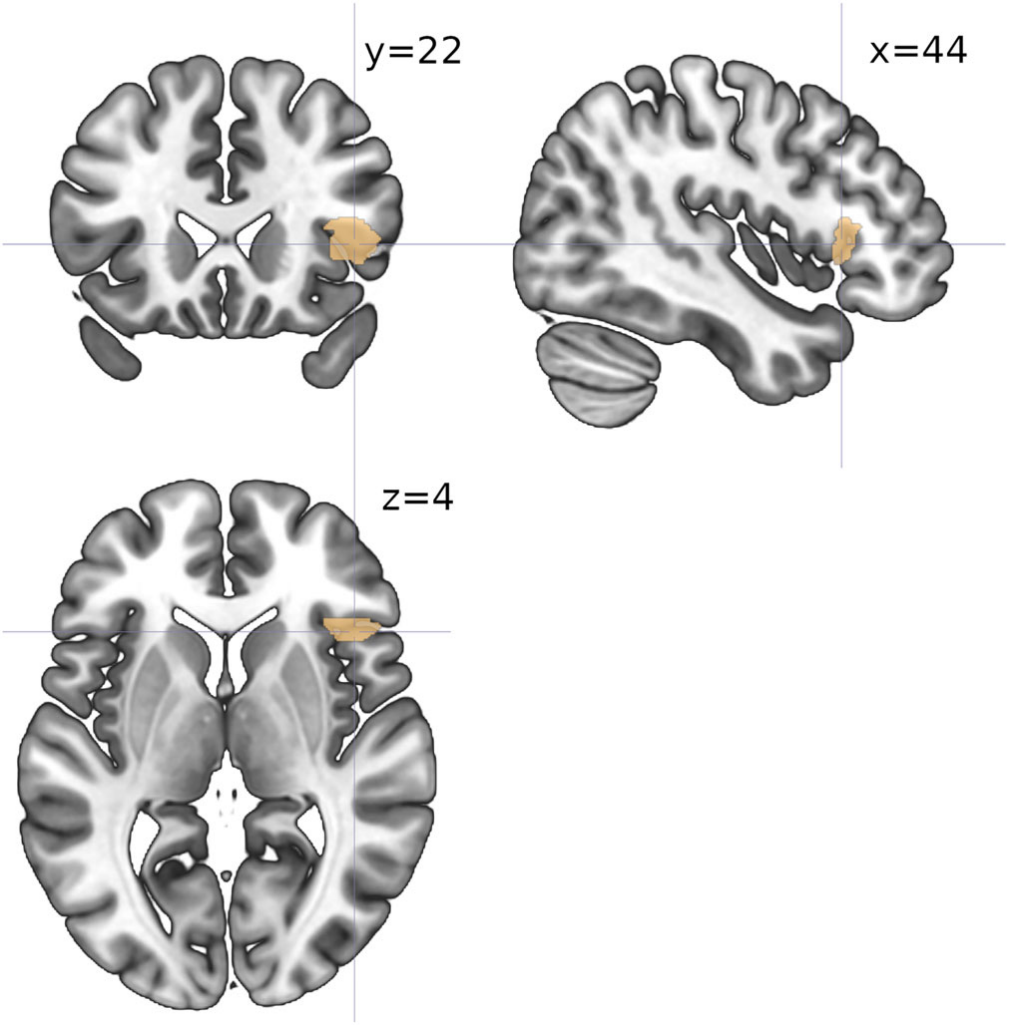
Activation map for the Seed‐based d‐Mapping with Permutation of Subject Images (SDM‐PSI) meta‐regression with prediction dummy variable {social = 1, nonsocial = 0}, for the positive side of the significance test. Cluster‐level inference with family‐wise error rate of *p* <.05

The SDM‐PSI meta‐analyses reported two surviving clusters for the SPE studies, and one for the NPE studies (Figure 5, Table 3). The SPE and NPE clusters included, but were not restricted to, the homologous clusters found via ALE; in particular, extensive bilateral medial frontal and anterior cingulate regions survived correction for both SPE and NPE, whereas caudate head and putamen activation was bilateral for NPE but left‐lateralized for SPE; finally, bilateral (but mostly left) insula activations survived only for SPE (Figure 5, Table 3). The SDM‐PSI meta‐regression yielded one surviving cluster, in the right inferior frontal gyrus, only for the positive tail of the {SPE = 1,NPE = 0} dummy variable, which marks a region where voxels covariated more when they belonged to SPE than to NPE studies (Figure 6, Table 3).

**TABLE 3:** Surviving Seed‐based d‐Mapping with Permutation of Subject Images (SDM‐PSI) meta‐analysis clusters

Analysis type	Cluster size (mm^3^)	Peak coordinates (MNI)	SDM‐*z* statistic	Local peaks		
				Coordinates (MNI)	SDM‐*z*	Anatomic location
SPE group				−4, 54, 4	8.190	Left superior frontal gyrus, medial, BA 10
				−38, 22, −12	6.712	Left inferior frontal gyrus, orbital part, BA 47
				−4, 10, −4	6.592	Left caudate nucleus, BA 25
	5479	−4, 54, 4	8.190	2, 42, 8	6.318	Right anterior cingulate/paracingulate gyri, BA 32
				0, 38, 6	6.003	Left anterior cingulate/paracingulate gyri, BA 32
				4, 38, 12	5.687	Right anterior cingulate/paracingulate gyri, BA 24
				−18, 8, −16	5.387	Left olfactory cortex, BA 48
				−36, 16, 0	5.347	Left insula, BA 48
				−32, 20, 2	5.033	Left insula, BA 47
				−12, 16, −8	4.798	Left striatum
				−2, 46, 18	4.702	Left superior frontal gyrus, medial, BA 32
				−2, 24, 54	4.385	Left supplementary motor area, BA 8
				2, 34, 24	4.384	Left anterior cingulate/paracingulate gyri, BA 24
				−22, 8, −6	4.117	Left lenticular nucleus, putamen, BA 48
				−2, 46, 36	3.996	Left superior frontal gyrus, medial, BA 9
				2, 24, 50	3.861	Left superior frontal gyrus, medial
				−2, 46, 32	3.798	Left superior frontal gyrus, medial, BA 9
				4, 28, −10	3.419	Right superior frontal gyrus, medial orbital, BA 11
				0, 26, −8	3.288	Left anterior cingulate/paracingulate gyri
				−2, 26, 40	3.277	Left superior frontal gyrus, medial, BA 32
				8, 32, −14	3.207	Corpus callosum
				−6, 26, −8	3.037	Left anterior cingulate/paracingulate gyri, BA 11
				2, 12, 42	2.862	Left median cingulate/paracingulate gyri
				26, 20, −6	5.472	BA 47
				38, 20, −8	5.356	Right insula, BA 47
				28, 16, −8	5.331	Right lenticular nucleus, putamen, BA 48
	621	26, 20, −6	5.472	28, 14, −4	5.296	Right lenticular nucleus, putamen, BA 48
				32, 0, −2	5.289	Right lenticular nucleus, putamen, BA 48
				44, 22, 2	4.305	Right insula, BA 47
NPE group				10, 8, −2	7.488	Right anterior thalamic projections
				4, 46, 0	6.916	Right superior frontal gyrus, medial, BA 10
				2, 52, 2	6.699	Right superior frontal gyrus, medial, BA 10
	6868	10, 8, −2	7.488	−2, 50, 2	6.647	Left anterior cingulate/paracingulate gyri, BA 10
				−18, 6, −6	6.422	Left striatum
				4, 8, −10	6.243	Right olfactory cortex, BA 25
				4, 8, −14	5.827	Right striatum
				18, 8, −8	5.656	Right striatum
				0, 46, 18	5.638	Left superior frontal gyrus, medial, BA 32
				34, −2, 4	5.614	Right lenticular nucleus, putamen, BA 48
				34, −8, 2	5.552	Right lenticular nucleus, putamen, BA 48
				0, 50, 16	5.340	Left superior frontal gyrus, medial
				−26, −2, −18	5.265	Left amygdala, BA 34
				−26, −8, −12	5.110	Anterior commissure
				−26, 2, −18	5.085	Left amygdala, BA 34
				14, 4, −8	5.061	Right striatum
				20, 0, −6	4.914	Right striatum
				10, 20, −12	4.784	Corpus callosum
				−20, 8, −20	4.779	Left inferior frontal gyrus, orbital part, BA 48
				−32, 0, −12	4.665	BA 48
				10, 12, −20	4.585	Right olfactory cortex, BA 25
				8, 14, −16	4.556	Right striatum
				−8, 56, −14	4.453	Corpus callosum
				16, 16, −12	4.361	Right inferior network, uncinate fasciculus
				−2, 44, −10	4.207	Left superior frontal gyrus, medial orbital, BA 11
				36, 0, 14	4.071	Right insula, BA 48
				26, −6, −14	3.887	Right inferior network, inferior longitudinal fasciculus
				8, 32, −14	3.693	Corpus callosum
				22, −2, −18	3.469	Right amygdala, BA 34
				−8, 34, −14	3.278	Left superior frontal gyrus, medial orbital, BA 11
				−26, −8, 0	3.079	Left pons
Meta‐regression {SPE = 1, NPE = 0} (positive)				44, 22, 4	2.916	Right inferior frontal gyrus, triangular part, BA 45
	174	44, 22, 4	2.916	48, 22, 2	2.647	Right inferior frontal gyrus, triangular part, BA 45
				54, 24, 8	2.107	Right inferior frontal gyrus, triangular part, BA 45
	2	48, 26, 18	2.278	48, 26, 18	2.278	Right inferior frontal gyrus, triangular part, BA 45
Meta‐regression {SPE = 1, NPE = 0} (negative)	No clusters survived					

Study heterogeneity, as measured via the statistic *I* ^2^, was 17.62%, 1.66%, and 34.45% for the NPE group, SPE group, and the linear meta‐regression, respectively. The funnel plots were reasonably symmetric for all three analyses; the metabias tests, which assess small‐study effects through funnel plot asymmetry, did not support the existence of small‐study effects (NPE: *z* = .76, *df* = 29, *p* = .45; SPE: *z* = 1.00, *df* = 26, *p* = .32; Meta‐regression: *z* = .30, *df* = 57, *p* = .77). Finally, there was no evidence for excess of significance (NPE: *p* = .91; SPE: *p* = 1.00, Meta‐regression: *p* = 1.00).

## 4 DISCUSSION

The ALE meta‐analysis suggested that SPE‐related activations were a subset of NPE‐related activations in the striatum. However, the SDM‐PSI meta‐analysis revealed a more complex picture, where extensive bilateral medial frontal, anterior cingular cortices, and striatal areas were shared between SPE and NPE (although striatal activations for SPE were rather left‐lateralized); and bilateral insular regions were found only for SPE studies. The meta‐regression found a single area in right middle ventrolateral PFC (Brodmann area 45 or BA45), predictive of more activation for SPE than NPE.

ALE did not find evidence in any brain region supporting a functional segregation of PEs with uncertainty originated in social versus nonsocial sources. The subtraction contrast SPE minus NPE was null, which suggests that social learning signals are a subset of general learning signals, and that value‐updating neural mechanisms are common to both social and nonsocial values. Although the nonsocial experiments selected covered a wider range of stimuli and task contexts (this does not affect the conclusion because by definition social contexts are excluded from the nonsocial condition) there were also no surviving clusters in the subtraction analysis NPE minus SPE (Table 2). However, absence of evidence is not evidence of absence. Not finding evidence for spatial segregation of learning signals via ALE could be due to the (insufficient) spatial resolution (~3 mm) or to the massive univariate approach typical of fMRI (Levorsen et al., 2021), if social‐specific mechanisms were finely segregated or distributed multi‐modally within regions recruited for both social and nonsocial tasks. Further, it is difficult to make a selection of learning signals (determined by the regressor used in the fMRI analysis design matrix) that is both liberal enough to encompass a sufficient amount of data, and restrictive enough not to thwart the validity of the results.

Since these considerations apply to any form of neuroimaging meta‐analysis, it is plausible that the difference between the ALE and SDM‐PSI results lie in their fundamentally different approach to statistical testing: because ALE only tests convergence of peaks, it is less sensitive to some effects that could be readily detectable through effect sizes. Conversely, by making use of the peak *t*‐statistics, SDM‐PSI can implement random effects modeling, which increases reliability and accuracy (Bossier et al., 2018). Thus, the significance of ALE and SDM‐PSI results should be considered in light of this subtle but important difference.

Overall, although most of the brain activations associated with learning error signals found were shared between social and nonsocial conditions, there was some evidence for functional segregation of error signals of exclusively social origin during learning in right BA45 in ventrolateral PFC (vlPFC) and insula. The right BA45 is a contralateral counterpart of Broca's area (left BA44 and BA45), which is active in semantic tasks. Neuroimaging studies have shown that the right vlPFC is a critical substrate of control (Levy & Wagner, 2011). Disrupting right vlPFC with repetitive transcranial magnetic stimulation impairs reasoning performance when logical conclusions are incongruent with beliefs, by impairing the inhibition of irrelevant information (Tsujii et al., 2010). The right vlPFC is also thought to be involved in re‐orienting attention to perceptual events that occur outside the current focus of attention (Corbetta et al., 2008) and in stopping and overriding motor responses, where BA45 is specifically associated with decision uncertainty (Levy & Wagner, 2011). Together with our findings, this hints that BA45 may not only respond to decision uncertainty, but more so to social than nonsocial decision uncertainty. At any rate, in line with previous research, striatal and medial frontal activations were conspicuous signatures of context‐independent signed PE signals (Bartra et al., 2013; Chase et al., 2015; Fouragnan et al., 2018; Levorsen et al., 2021; McClure et al., 2003). Drawing from our results and extant literature, we propose a parsimonious scheme that builds on the common currency hypothesis (Levy & Glimcher, 2012) that goes beyond the distinction social/nonsocial to account for learning, storage, and retrieval of values in the brain.

### 4.1 The hourglass schema: a common currency bottleneck

**FIGURE 7:**
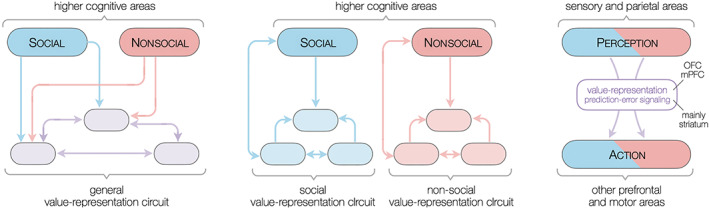
Candidate neural architectures subserving social and nonsocial value representation and learning. Extended common currency schema (left), social brain or social valuation‐specific schema (center), and hourglass schema (right). The extended common currency schema and social valuation‐specific schemas are based on Ruff and Fehr's (2014) homologous schemata.

Value representation occurs primarily *within* ventral and medial PFC, where it is segregated to varying extent by categories (Clithero & Rangel, 2013) that include a distinction between social and nonsocial (Grabenhorst & Rolls, 2011; Lieberman et al., 2019). Using a meta‐analytic approach, we found that learning‐related areas were shared between social and nonsocial conditions, except perhaps for some evidence for social‐nonsocial segregation in the form of left‐lateralized social error signals in the striatum. This is consistent with the notion that the striatum is a general‐purpose subcortical region capable of integrating social information into coding of social action and reward (Baez‐Mendoza & Schultz, 2013; Klucharev et al., 2009; Rilling et al., 2004). In contrast, the preceding and subsequent stages in the stimulus‐decision pipeline—perception and action—are functionally specialized for different stimuli and motor commands. This suggests a schema where value representation and learning circuits lie at a bottleneck in the information stream flowing from stimulus to action, and the degree of localized specialization is proportional to the distance to the bottleneck (Figure 7, right). The existence of persistent common‐currency value representations in mPFC/OFC (Levy & Glimcher, 2012) is motivated by their postulated privileged location atop the cortical hierarchy of nested time scales (Hasson et al., 2015; Kiebel et al., 2008; Murray et al., 2014). This means that values stored in mPFC/OFC regions are among the longest lasting stored representations in the brain. The hourglass schema posits that values learned through repeated interaction with the world are located near or at the top of the cortical hierarchy, and that value representation and updating mechanism for categories such as social and nonsocial are segregated only locally within mPFC/OFC or striatum respectively, but that a single neural circuit performs the computation of value for both social and nonsocial events. Thus, mPFC/OFC would be a module of common currency values (comprising sets or hypercolumns of common currency values for different objects), that are routinely accessed for action selection and perception and updated after feedback. This contrasts with the social brain hypothesis, where social aspects of the environment are processed in a neural circuitry that evolved specifically to deal with them (Ruff & Fehr, 2014), which entails that social and nonsocial values are functionally segregated in the brain (Figure 7, center). On the other hand, the hourglass schema—with its common currency representation (in mPFC/OFC) and updating (striatum) at the bottleneck of the stimulus‐value‐action pipeline of value‐based decision‐making—is similar to the extended common currency schema (Ruff & Fehr, 2014) which assumes that a single modular neural circuit with common reward‐related areas values both social and nonsocial events (Figure 7, left).

Consistent with the thesis that value‐based learning processes are not functionally segregated by category, Behrens et al. (2008) proposed that social information was acquired using the same processes as general associative learning: two neighboring divisions of the ACC were implicated in parallel learning of social and nonsocial information and in using it to guide behavior, with the ventromedial prefrontal cortex (vmPFC) merging evidence from both divisions to make a decision. This concurs with the surviving clusters we found in both ALE and SDM‐PSI analyses. They also found that the middle temporal gyrus (MTG), the right superior temporal sulcus (STS), temporoparietal junction (TPJ), and dorsomedial prefrontal cortex (dmPFC) were involved in social valuation. The only surviving clusters in the subtraction or meta‐regression SPE‐NPE was a subregion of ventrolateral PFC (vlPFC), which suggests that social‐specialized mechanisms modulating behavior are not part of the valuation bottleneck in vmPFC/OFC. Thus, in line with studies suggesting that the learning of social values reuses the general‐domain striatal value‐updating mechanisms (Behrens et al., 2008; Grabenhorst & Rolls, 2011; Zhang & Gläscher, 2020), we conclude that differences in how brains learn in social contexts occur before and after the value representation stage, and that the value‐updating hub in the striatum deals in a centralized manner with learning value, analogously to how the mPFC/OFC comprises the neural representation of a common currency for value (Levy & Glimcher, 2012; Ruff & Fehr, 2014). This leads us to suggest that functional specialization in learning values occurs outside the common currency regions OFC and mPFC, via their efferent connections to other brain areas segregated by social context, that presumably can modulate learning through cortico‐striatal loops.

vmPFC/OFC sends projections to reach cingulate cortex and adjacent prefrontal areas, receives afferents from striatum, and has bidirectional connections with the temporal lobe (Carlson, 2013; Price, 2006). Thus, many PFC regions that have been attributed a role in social cognitive and affective, and mentalizing functions might do so by tapping into the neighboring vmPFC/OFC hosting value representations (see summary points of Rilling & Sanfey, 2011). For example, the anterior rostral medial frontal cortex (amPFC) has been proposed to subserving mentalizing computations about one's own and other's mental states (Amodio & Frith, 2006). A study restricted to the mPFC (Lieberman et al., 2019) that, via a multi‐domain approach that enabled making causal inferences, found evidence for social processes being linked to the vmPFC and dorsomedial prefrontal cortex (dmPFC), self‐related processes to the amPFC, and complex contextual processing to vmPFC. Thus, both social and general domain values, experienced pleasure, and expectations might be represented under a common value currency in the vmPFC/OFC from where they are relayed to ACC and posterior frontal areas to guide action selection (Grabenhorst & Rolls, 2011). In summary, there is evidence supporting some degree of local functional segregation of social and nonsocial value representations, but the value representation and learning algorithms seem to be shared.

### 4.2 Are there social‐specialized neural mechanisms?

The thesis of a centralized scheme for value computation clashes with studies standing behind social‐specialized neural mechanisms (Allison et al., 2000; Amodio & Frith, 2006; Brass et al., 2007; Frith & Frith, 2012; Gallagher & Frith, 2003; Krajbich et al., 2009; Levorsen et al., 2021; Lombardo et al, 2011; Samson et al., 2004; Saxe & Wexler, 2005; Stone et al., 2002).

The evolutionary precursor of (social) cognition is sensorimotor interaction of creatures with the environment (Varela et al., 1992): the actions that creatures are capable of executing condition the form of its perceptual apparatus, and vice versa. This is particularly conspicuous in reactive environments (e.g., social) where the consequences of actions are fraught with high uncertainty and potentially fatal. Since human ancestors maneuvered social environments for millions of years, motor control, perception, and social interactions are likely to be at heart inextricably intertwined processes (Wolpert et al., 2003), which in general cannot be understood separately (e.g., control and perception of facial and manual expressions; Iacoboni et al., 2005; Peelen & Downing, 2007; Todorov et al., 2008). Although this does not imply that the neural processes subserving social interactions are not specialized, most of the brain regions reported to be activated during social decision‐making are also recruited by nonsocial decision making; these areas are chiefly PFC, ACC, anterior insula, ventral striatum, and amygdala (Rilling & Sanfey, 2011).

Perhaps the most plausible neural circuits associated with social decision‐making that are candidate to social‐specificity are those involved in theory of mind, especially in the self‐other distinction. Some of the functional specialization of social representations reported in the literature could spring from self versus other differences. Baez‐Mendoza & Schulz (2013) reviewed the role of striatum in encoding reward related information both to self and others' actions. For self actions*, s*triatal activity relates to movements' initiation and execution (self‐initiated, ordered, or both; Hollerman et al., 2000; Schultz & Romo, 1988; Romo et al., 1992), reward receipt and expectation (Apicella et al., 1992; Hikosaka et al., 1989; Schultz et al., 1997), and actions that lead to reward (Kimchi et al., 2009; Kimchi & Laubach, 2009) or not (Hollerman et al., 1998; Kawagoe et al., 1998), conjunction of reward and actions, and reward‐predicting cues (in caudate, Kawagoe et al., 1998; Lauwereyns et al., 2002). For others' actions, Klein and Platt (2013) showed reward type selectivity of striatal neurons and substructures: stronger modulation of caudate by social, and of putamen by nonsocial rewards. Although vicarious reward in observational learning (learning from other person acting and receiving reward) is distinguished from “pure” social‐reward (an actor is rewarded by conspecifics) and from observing‐reward (observing is rewarding itself), all engage striatal neurons (Klein & Platt, 2013; Moll et al., 2006). During observation of others, learning action‐value comes not from direct reward, but is based on “action observation prediction error” (dlPFC) and “outcome observation prediction error” (vmPFC positive correlation, ventral striatum negative correlation; Burke, Tobler, Baddeley, et al., 2010; Burke, Tobler, Schultz, et al., 2010). In a task where subjects learned to predict behavior of conspecifics, Suzuki et al. (2012) reported that observation of others' choices generated simulated‐other's action PE encoded in dmPFC/dlPFC, and a simulated‐other's reward PE processed in vmPFC. To sum up, while there are some hints of specialization between self‐ and other‐centered representations, the core substrate is mostly shared.

Foremost regions ascribed to the neural substrates of theory of mind are mPFC, superior temporal sulci, and temporal poles as “theory of mind hubs” (Gallagher & Frith, 2003), precuneus and TPJ for mentalizing (especially right TPJ, Frith & Frith, 2012; Saxe & Wexler, 2005), dmPFC for others' beliefs (Jamali et al., 2021), and STS for biological motion (Allison et al., 2000; Brass et al., 2007). The strongest evidence for social specialization comes from neurology and psychiatry, where it is possible to make causal inferences about the role of particular brain regions. Social anxiety, autism, and bipolar disorders are linked to abnormalities in social stimulus‐evoked activations in striatum (Sripada et al., 2013), ACC (Chiu et al., 2008), and insula (King‐Casas et al., 2008), respectively. Research on autism suggests that mentalizing is subserved by specialized neural mechanisms localized in right TPJ, that can be selectively impaired (Lombardo et al., 2011). The social origin of this abnormality is supported by patients with autism being insensitive to social reputation (Izuma et al., 2011). Stroke‐related lesions can strongly affect social aspects of behavior: extensive damage to OFC, temporal poles, and amygdala, selectively impairs social reasoning after controlling for formally similar nonsocial problems (Stone et al., 2002); damage to vmPFC is associated with lack of concern for others and insensitivity to guilt (Krajbich et al., 2009) and with modulating behavior by accounting for social aspects in the ultimatum game (Moretti et al., 2009); and left TPJ is necessary for representing others' beliefs (Samson et al., 2004). It transpires that the loss of some brain regions entails the loss of some social skills, but not of their nonsocial analogues. Importantly, social factors seem to modulate how social stimuli are represented (frequently through TPJ and other temporal areas)—thereby potentially modulating learning‐related striatal activations (Sripada et al., 2013)—and action selection in social contexts (through mPFC and ACC), but the subcortical structures elicited by error signals are not specific to social information.

### 4.3 A common currency of value exchanged between specialized representational and strategic action modules

The emerging hazy picture of social decision making comprises a valuation stage that is shared with nonsocial decision making, and a constellation of associative and executive modules that are at least in part specific to some social stimuli and contexts. The most conspicuously specialized modules are those involved in theory of mind and self‐other distinction, and are localized in the temporal lobe. However, even the existence of regions that are causal effectors of theory of mind does not imply functional specialization in theory of mind (or in general in social tasks). This is similar to the distinction between uniformity and association test maps on the Neurosynth database (Yarkoni et al., 2011; the association test map with the keyword “social” on Neurosynth yields the brain regions temporal pole, precuneus, dmPFC, vmPFC/OFC, ACC, TPJ, amygdala, insula, in agreement with the studies reviewed here). Although some regions are considered to be specifically associated with social domains, this could be explained by social environments being typically more complex (e.g., due to being noisier) than their nonsocial counterparts: many neuroimaging studies have found that social interactions with human players produce stronger activations than similar interactions with computer players in several “social” brain areas (Lee, 2008). Thus, “social” areas are more likely to subserve high‐level general domain computational modules, that are conspicuously recruited in social situations, which are highly demanding of recursive and strategic reasoning, usually through noisy inferences about other's intentions (Iacoboni et al., 2005). In summary, although social decision‐making recruits many areas (Rilling & Sanfey, 2011), those areas are unlikely to be specific to social decision‐making. This is plainly articulated by Stone and Gerrans (2006): “Lower‐level domain‐specific mechanisms … interacting with higher‐level domain‐general mechanisms for metarepresentation, recursion, and executive function can account for observed patterns of deficits in both autism and neurological patients.” Therefore, we suggest that while adaptive perception and behavior are context, stimulus, and goal dependent—and thus modulated by social factors—the most persistent and affective values that drive behavior have a common substrate in OFC/vmPFC that serves as a hub for value retrieval and updating, mainly through its connections with temporal, adjacent prefrontal, and subcortical regions. For example, the right TPJ often encodes socially relevant states involving theory of mind (Frith & Frith, 2012; Saxe & Wexler, 2005) that together with contextual information from ACC and experiential values in vmPFC enable the integration and generation of learning signals (Zhang & Gläscher, 2020); further, in social interactions involving gauging the influence of others' rewards, social value could be converted to the common currency value (Fukuda et al., 2019) such that others' values encoded in right TPJ and left dlPFC are combined with insula activity to yield final decision value in mPFC; finally, our results also suggest that vlPFC (BA45) responds to decision uncertainty, but more so in social than in nonsocial learning contexts.

## 5 CONCLUSION

We suggest that mechanisms involved in learning itself are not specific to social preferences or social uncertainty, and neural modules specialized in the representation of the multiple facets of complex social environments are distributed mainly in temporal and medial prefrontal areas. Notably, these modules are not exclusive to social concepts, and some are more strongly and frequently activated by social than nonsocial situations, such as those involved in theory of mind. This entails that higher‐order association areas such as TPJ and rostral temporal cortex, and strategic action selection areas such as mPFC and vlPFC are the most plausible candidates for regions functionally specialized in the representation and deployment of social preferences (Amodio & Frith, 2006; Behrens et al., 2008; Coricelli & Nagel, 2009; Levy & Wagner, 2011; Saxe & Wexler, 2005; Zhang & Gläscher, 2020). This is supported by evidence that social preferences and states are represented in multiple distributed brain areas in the form of self‐referential, attitudinal, affective, and other uncertain and internally generated social variables (Mitchell, 2009). Further, this also suggests that the loci devoted to learning from error signals are likely to constitute a flexible and general‐purpose learning system (for a common currency of value) whose hub sits in the dopaminergic mesocortical pathway. Although learning and action selection in social contexts are typically complex and higher‐level cognitive process invariably requiring explicit cognitive control, their underlying primary infrastructure is supported by general‐purpose mechanisms in the basal ganglia (Hélie et al., 2015). Thus, although explicit metacognition presumably evolved to enhance social skills in a relatively short time span (Fletcher & Carruthers, 2012; Frith, 2012), these adaptations are ostensively concerned with value representation, as opposed to with PE signaling, and thus are restricted to association and strategic areas in the neocortex.

There are other conceivable explanations for our not finding more evidence of dissociation between learning under conditions of social and nonsocial uncertainty. First, we focused specifically on error signal events that occur exclusively during feedback; this precluded the detection of many putative learning mechanisms occurring after feedback. Second, our focus on uncertainty of social origin *and* on learning mechanisms severely limited the number of eligible studies, compared with similarly‐themed meta‐analyses. For example, Chase et al. (2015) conducted a meta‐analysis of all tasks encompassing RL modeling, thus bringing to bear a broader selection of fMRI studies. However, their definition of PE was narrower, since we regarded as PE any learned variable resting on interactions with a conspecific, regardless of whether subjects were modeled as RL algorithms. Also, their definition of SPE was predicated on the content of reward itself (social or nonsocial), whereas ours was predicated on the source of noise. This is presumably the cause underlying the disparity between results; such as the absence of frontal operculum and insula regions in the subgroup analyses. Third, since BOLD signals reflect current input similarly to local field potentials (Logothetis, 2008), it is possible that there exist more areas functionally segregated by sociality from which efferent signals contribute to the learning signals found in the striatum. However, the literature suggests that contributions from regions affording contextual or value signals are likely to be from regions not directly involved in learning, but in temporally persistent representations, that can modulate learning, like TPJ (Zhang & Gläscher, 2020).

In conclusion, most of the neural circuitry in value learning and representation regions was not segregated into distinct modules processing uncertainty (noise) of social versus nonsocial origin; however, we found some evidence supporting functional specialization in insula for social error signals and in vlPFC for being differentially more activated in social than in nonsocial contexts. This suggests that most behavioral adaptations to navigate social environments are reused from frontal and subcortical areas along the mesolimbic pathway processing generic value representation and learning, but that specialized structures might have evolved in the prefrontal cortex to deal with social context representation and strategic action. This has implications for social, developmental, and evolutionary neuroscience, because it suggests that the mesolimbic pathway could have been reused and deployed, with little modification, to serve the mounting computational needs of human ancestors' brains in an increasingly complex social environment.

## CONFLICT OF INTEREST

The authors declare no conflict of interest.

## Data Availability

The authors confirm that the data supporting the findings of this study are available within the article.
